# Attitudes and perceived barriers to firearm safety anticipatory guidance by pediatricians: a statewide perspective

**DOI:** 10.1186/s40621-021-00319-9

**Published:** 2021-09-13

**Authors:** Bijan Ketabchi, Michael A. Gittelman, Hayley Southworth, Melissa Wervey Arnold, Sarah A. Denny, Wendy J. Pomerantz

**Affiliations:** 1 Department of Pediatrics, University of Cincinnati College of Medicine, Division of Emergency Medicine, Cincinnati Children’s Hospital Medical Center, 3333 Burnet Avenue, ML #2008, Cincinnati, OH 45229 USA; 2 Ohio Chapter of the American Academy of Pediatrics, 94-A Northwoods Blvd., Columbus, OH 43235 USA; 3 Division of Primary Care Pediatrics, Nationwide Children’s Hospital, 700 Children’s Drive, Columbus, OH 43205 USA

**Keywords:** Firearm safety, Anticipatory guidance, Barriers, Pediatrician attitudes

## Abstract

**Background:**

Firearms are the second leading cause of injury-related death in American children. Safe storage of firearms is associated with a significantly decreased odds of firearm-related death, however more than half of US firearm owners store at least one firearm unlocked or accessible to a minor. While guidance by primary care providers has been shown to improve storage practices, firearm safety counseling occurs infrequently in the primary care setting. The primary objective of this study was to describe pediatricians’ perceived barriers to providing firearm safety education to families in the pediatric primary care setting. Secondary objectives included identifying pediatric provider attitudes and current practices around firearm counseling.

**Methods:**

This was a cross-sectional survey of pediatric primary care providers in Ohio. Participants were recruited from the Ohio AAP email list over a 3-month period. Only pediatric primary care providers in Ohio were included; subspecialists, residents and non-practicing physicians were excluded. Participants completed an anonymous online survey detailing practice patterns around and barriers to providing firearm safety counseling. Three follow-up emails were sent to pediatricians that failed to initially respond. Response frequencies were calculated using Microsoft Excel.

**Results:**

Two hundred eighty-nine pediatricians completed the survey and 149 met inclusion criteria for analysis. One hundred seven (72%) respondents agreed that it is the responsibility of the pediatric primary care provider to discuss safe storage. Counseling, however, occurred infrequently with 119 (80%) of respondents performing firearm safety education at fewer than half of well child visits. The most commonly cited barriers to providing counseling were lack of time during office visits, lack of education and few resources to provide to families. A majority, 82 of pediatric providers (55%), agreed they would counsel more if given additional training, with 110 (74%) conveying they would distribute firearm safety devices to families if these were available in their practice.

**Conclusion:**

Ohio pediatricians agree that it is the responsibility of the primary care provider to discuss firearm safety. However, counseling occurs infrequently in the primary care setting due to a lack of time, provider education and available resources. Improving access to resources for primary care pediatricians will be critical in helping educate families in order to protect their children through improved storage practices.

## Learning objectives

1. Understand attitudes of primary care pediatricians about counseling families on firearm safety during well-child visits.
2. Learn about current practices of pediatricians in regard to counseling families about firearm safety.
3. Identify the main barriers to firearm safety counseling in the primary care setting and potential ways to overcome them.

## Background

Firearms remain a leading cause of morbidity and mortality in American children (Grinshteyn and Hemenway 2016; WISQARS Fatal Injury Reports 2017) accounting for the deaths of nearly 1300 children (age < 18 years) annually (Fowler et al. 2017). Deaths from firearms account for roughly 15% of all childhood deaths; comparable to the annual deaths from motor vehicle crashes nationwide (WISQARS Fatal Injury Reports 2017). Fortunately, the risk of suicide and unintentional injury by firearm can be greatly mitigated with safe storage of firearms—by as much as 78% (Grossman 2005). Safe storage of firearms is defined as weapons stored locked, unloaded and with ammunition locked and stored separately (Grossman 2005). Unfortunately, data show that the majority of families do not store their firearms safely (Albright and Burge 2003; Baxley and Miller 2006; Barkin et al. 2008). A recent study from the Journal of the American Board of Family Medicine reported that only 36% of families with firearms store them safely (Albright and Burge 2003) corroborating data from another study which found that 73% of children under the age of 10 knew the location of a firearm in the home, and 36% admitted to personally handling it (Baxley and Miller 2006).

Anticipatory guidance and safety counseling are key aspects of the well child visit. Primary care pediatricians regularly provide injury prevention counseling on many topics including bicycle helmets, motor vehicle safety, and water safety (WISQARS Fatal Injury Reports 2017). In fact, we know that when physicians counsel about firearm safety, families improve their safety behaviors (Albright and Burge 2003; Barkin et al. 2008). In one study it was reported that 1 out of every 2.5 patient-families practiced safer firearm storage after verbal safety counseling from a pediatrician or nurse practitioner during well child visits (Barkin et al. 2008). While physician counseling has been shown to be effective in producing safer storage practices (Barkin et al. 2008), only a small minority of providers regularly counsel about firearm safety (Damari et al. 2018). A recent study published in the American Medical Association Journal of Ethics details that despite the majority of physicians knowing how to counsel about firearm safety “only 25 percent reported having conversations with patients about firearms or firearm safety often or very often” (Olson et al. 1997). When comparing the frequency of firearm counseling relative to other topics, such as driving safety, these other subjects appear to be covered much more regularly. One study of 160 pediatricians, revealed that 93 and 89%, regularly counseled about seat belt use and impaired driving, respectively, at well child visits. These numbers reveal a vast discrepancy in the frequency of counseling of these issues, despite the fact that firearm-related deaths are on par with the scale of motor vehicle-related deaths in many states across the country.

While there have been some studies examining physicians’ attitudes toward firearm safety counseling (Solomon et al. 2002), to our knowledge, there have been only a few that focus on pediatricians (Hoops and Crifasi 2019; Beidas et al. 2019; Campbell et al. 2009). It is critical to better understand the specific barriers faced by pediatricians as childhood firearm suicides have increased by almost 90% over the last 10 years (WISQARS Fatal Injury Reports 2017). Of these pediatric studies, some were conducted more than 15 years ago (Hoops and Crifasi 2019; Beidas et al. 2019) and may not reflect attitudes of today, while others solely focus on pediatric residents (Beidas et al. 2019; Campbell et al. 2009) rather than practicing primary care providers. What almost all prior studies fail to address are barriers that physicians may encounter in providing this counseling. The purpose of this study was to identify pediatric primary care provider attitudes, anticipatory guidance practices and perceived barriers to providing firearm safety education for families in the pediatric primary care setting within one state.

## Methods

### Survey development and content

An electronic survey was created using Survey Monkey**®** software. As no validated surveys exist to address this type of barrier, this survey content was formulated by a panel of physicians specializing in injury prevention and experts in survey tool development. The survey contained 19 questions and collected information including: demographics, practice environment, attitudes toward firearm counseling, self-reported screening/counseling practices, perceived barriers to discussing firearm safety, and availability of resources/training. Pediatric providers were asked to separately describe their frequency of screening and counseling families of young children (age < 13) and families with teens using multiple choice questions divided into ten percentile ranges. Barriers included in the multiple-choice responses were decided a priori using expert opinion and previous studies as a framework (Olson et al. 1997; Solomon et al. 2002; Hoops and Crifasi 2019). The questions were piloted among several pediatricians prior to distribution to evaluate clarity and survey content.

### Study protocol

The Ohio Chapter of the American Academy of Pediatrics (AAP) has more than 2900 pediatricians within their database. Of these, 1539 self-identified as a primary care provider (PCP) when they signed up with the AAP. After the study received approval by the IRB, the survey was distributed to these primary care pediatricians via email. A cover letter describing the purpose of the survey and the reason the recipient was chosen to participate was sent to all selected providers. To be included in the study, participants had to have completed a residency in pediatrics and identify themselves as a PCP in Ohio. Those whose roles in injury prevention counseling may be unclear, variable, or directed by a governing body (e.g. a residency program)—such as non-clinical providers, sub-specialists, or trainees —were excluded. Participants were also excluded if they had a non-working or no email address. Survey completion was assessed through Survey Monkey®; answers from incomplete surveys were included in final analysis. These pediatricians were asked questions regarding the frequency of screening for and counseling about firearm safety in addition to selecting the three most common barriers they encounter when broaching this subject. The first email was sent on October 1, 2018 to 1539 members of the Ohio AAP. Two follow-up invitations were sent three weeks apart to those who had not previously completed the survey. The first follow-up was sent on October 24, 2018 to 1509 members; the 30 members who had previously responded and provided contact information were removed. The final survey was sent on November 30, 2018 to 1487 members, again removing those who responded and provided contact information.

### Data analysis

The survey was divided into sections: Demographics, Attitudes and Current Practices, and Barriers to Providing Counseling. The survey items were grouped based on age range of the patients in the questions in order to provide more clarity for participants. Survey results were exported from Survey Monkey into Microsoft Excel. From there, Excel functions were used to quantify frequencies for each item of the survey as well as create graphical displays and tables to for our results. Frequencies were calculated for each item in the survey.

## Results

### Demographics

Of the 1539 PCPs in Ohio, 289 (19%) surveys were completed. The final sample included 149 participants. Of the 140 excluded from our analysis, 6 did not complete a Pediatric residency, 122 did not self-identify as a PCP, and 12 did not meet either criteria. Eighty one participants (64%) were female. The largest percentage of respondents (*n* = 37, 29%) were 45 to 54 years of age. Nearly half (*n* = 58, 46%) practiced in a suburban setting, while 42 (34%) and 25 (20%) practiced in urban and rural settings, respectively. Demographic results are summarized in Table 1. When compared to overall composition of Ohio AAP membership, our participants are representative of the group with regard to gender and age distribution.

**Table 1:** Participant demographics

*N* = 149	Response	Number (%)	Chose Not to Respond
Gender	Female	81 (64%)	*N* = 23
	Male	43 (34%)	
	Prefer not to answer	2 (2%)	
Age	18 t 24	1 (1%)	N = 23
	25 to 34	17 (13%)	
	35 to 44	24 (19%)	
	45 to 54	37 (29%)	
	55 to 64	29 (23%)	
	65 or older	16 (13%)	
	Prefer not to answer	2 (2%)	
Practice Setting	Rural	25 (20%)	*N* = 24
	Suburban	58 (46%)	
	Urban	42 (34%)	

### Attitudes toward firearm safety counseling

**Fig. 1:**
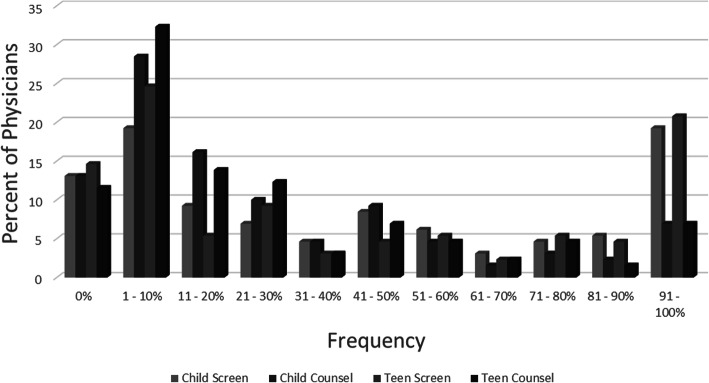
Self-reported frequency of screening for firearm ownership and counseling about firearm safety at well child visits with families of young children and families of teens

While a majority of respondents (72%) “Agreed” or “Strongly Agreed” that “It is the responsibility of the physician to discuss firearm safety”, screening for and counseling about firearm ownership occurred infrequently. Overall, 61.5% of pediatric primary care providers reported screening for firearm ownership at less than half of well child visits. Among families of young children (age < 13), 81.5% counseled less than half the time, with 67.6% counseling in less than 30% of visits. Regarding families of teens, 80% of pediatricians performed counseling at less than half of well child visits, with 70% of providers doing so less than 30% of the time. (Fig. 1).

### Barriers toward firearm safety counseling

The most commonly cited barriers to providing counseling was “lack of time” (70%), followed by “lack of resources” (24%) and “fear of negative reaction” (23%). (Table 2) The least commonly cited barriers included: “Do not believe this is relevant to my patients/practice setting” (10%), and “Do not believe it is the duty of the primary care physician” (< 2%). Only 23 (18%) pediatricians felt they had adequate materials to distribute to patients and their families. Furthermore, just 49 (39%) respondents felt they had received adequate training to properly counsel families about firearm safety. When asked about changing their current practices, 55% of pediatricians responded that they would counsel more frequently if given additional training and/or resources. Additionally, 74% would distribute safety devices to patients and their families if these resources were provided to their practice.

**Table 2:** Perceived barriers to providing anticipatory guidance. Each provider selected up to three responses

Barriers to Providing Anticipatory Guidance	Percent Citing Each Factor
Limited time	70.1
Lack the resources necessary	23.6
Fear of negative reaction	22.8
Not part of routine WCC	21.2
Not familiar enough	20.5
Do not believe counseling changes behavior	17.3
Do not have any barriers	15.7
Only discuss if at risk	12.6
Do not believe relevant to my patients/practice setting	10.2
Other	7.1
Do not believe it is duty of PCP	1.6

## Discussion

Our study is among the first to examine pediatricians’ attitudes toward and frequency of firearm safety counseling. Moreover, our study is the first to identify the specific key barriers faced by primary care pediatricians when providing firearm-related anticipatory guidance. This cross-sectional study demonstrates that while the majority of Ohio pediatric primary care providers believe that it is their responsibility to discuss firearm safety at well child visits, most fail to counsel consistently.

The most commonly cited barriers to providing this counseling are limitations in time and resources. We found that the majority of respondents agreed that they would indeed counsel more frequently if given access to additional resources, such as firearm safety locks or educational material. Our findings corroborate with previous studies pointing toward lack of education as a key hindrance to providing firearm safety counseling (Hoops and Crifasi 2019). Pediatricians also cite similar barriers when counseling about other preventive topics during well child visits, such as drug abuse counseling (Kulig 2005). However, the one barrier that appears to be unique in discussing firearm safety is the citing of lack of resources for providers. It should also be noted that our third most cited barrier, “fear of negative reaction”, has been examined previously and three quarters of parents agreeing that pediatricians should discuss firearm safety (Garbutt et al. n.d.).

In order to overcome these barriers, education is key—for both families and pediatric providers. Medical students and pediatric residents have a core curriculum that they are taught throughout their training. One way of improving their ability to counsel effectively about firearms could be to incorporate more firearm-based anticipatory guidance into this curriculum, which has been shown to improve self-efficacy but likely requires sustained education (Kwong et al. 2019). Furthermore, our study also illustrates that the majority of pediatricians do not feel they have access to an adequate resources to provide to families, but would likely counsel more if they did. One resource that is available to pediatricians is the Ohio AAP’s “one-pagers” about firearm safety. On the Ohio AAP’s website, educational handouts are available for both physicians and families. Previous studies that examined office-based interventions have indicated that when physicians provide families with tangible resources, such as safety locks, a large number of families will put these into use (Barkin et al. 2008). Making a change toward safer storage is key to decreasing suicides and unintentional injuries among the pediatric age group (Monuteaux et al. 2019). Future projects aimed at providing primary care pediatricians with crucial resources, such as safe storage devices, may result in not only increased counseling frequency, but also significant decreases in firearm-related morbidity.

In the setting of a relatively low response rate, one challenge is determining if those responding are representative of Ohio pediatricians as a whole. Responders may be more invested in the topic or significantly different (e.g. more likely to provide counseling) in the variables of interest than those who did not respond; however, our study’s response rate is not unlike those of similar physician surveys (Campbell et al. 2009).

### Limitations

Our study was limited by the relatively small sample size, but our response rate was similar to that of other surveys distributed to the Ohio AAP. Nonresponse bias is a major consideration in a study such as this. In order to mitigate this multiple waves of surveys were distributed with repeated outreach to those specifically who had not responded. While analysis of the non-responders is not possible, it is probable that these non-responders differed significantly in their practices from responders; both in frequency of counseling and barriers face. Future qualitative studies, such as telephone calls to non-responders, may be able to aid in understanding the degree of participation bias. Given this sample size, we were not powered to compare frequencies among different subpopulations of physicians such as age. Our study was conducted only among Ohio Pediatricians, results may vary significantly based on other states’ frequency of firearm ownership or other yet-to-be-determined factors. As with all surveys, our study contains the potential for recall bias as all frequencies were self-reported. Despite these limitations, our study has helped elucidate a deficiency in the primary care setting.

## Conclusions

Our study substantiates previous reports that, while primary care pediatricians feel it is their duty to discuss firearm safety, this counseling occurs infrequently. By helping to identify some of the most common barriers to providing counseling, our study hopes to lay the groundwork for targeted interventions focused on addressing these key issues. Our findings show that the majority of respondents feel they would counsel more regularly if they had access to more resources. Future studies will examine if additional time during well child visits or supplementary funding for resources, such as firearm locks, results in increased counseling frequency and safe storage.

## Data Availability

All data is maintained at Cincinnati Children’s Hospital Medical Center on password protected computers. The datasets generated and/or analyzed during the current study are not publicly available due containing identifying/contact information, but are available from the corresponding author on reasonable request.

## Abbreviations

AAP: American Academy of Pediatrics
IFCK: Injury Free Coalition for Kids
PCP: Primary care provider
